# Glypican 4 Regulates Aβ Internalization in Neural Stem Cells Partly *via* Low-Density Lipoprotein Receptor-Related Protein 1

**DOI:** 10.3389/fncel.2021.732429

**Published:** 2021-09-06

**Authors:** Kaige Ma, Shan Xing, Yan Luan, Chenglin Zhang, Yingfei Liu, Yulang Fei, Zhichao Zhang, Yong Liu, Xinlin Chen

**Affiliations:** ^1^Institute of Neurobiology, Department of Neurobiology, Xi’an Jiaotong University Health Science Center, Xi’an, China; ^2^Department of Neonatology, Children’s Hospital Affiliated to Zhengzhou University, Henan Children’s Hospital, Zhengzhou Children’s Hospital, Zhengzhou, China; ^3^2018 Grade, Zonglian College, Xi’an Jiaotong University Health Science Center, Xi’an, China; ^4^Medical College, Xijing University, Xi’an, China

**Keywords:** glypican 4, neural stem cells (NSCs), low density lipoprotein receptor related protein 1, β-amyloid internalization, Alzheimer’s disease

## Abstract

Neural stem cell (NSC) damage has been reported in patients with Alzheimer’s disease. Intracellular Aβ plays a vital role in NSC damage. Heparan sulfate proteoglycans are potent mediators of Aβ enrichment in the brain. We hypothesized the heparan sulfate proteoglycan glypican 4 (Gpc4) regulates Aβ internalization by NSCs. We evaluated Gpc4 expression in NSCs from P0–P2 generations using immunofluorescence. Adenovirus and lentivirus were used to regulate Gpc4 expression in NSCs and APP/PS1 mice, respectively. Co-immunoprecipitation was used to determine the relationship between Gpc4, Aβ, and low-density lipoprotein receptor-related protein 1 (LRP1). Intracellular Aβ concentrations were detected using enzyme-linked immunosorbent assay and immunofluorescence. The role of Gpc4/LRP1 on toxic/physical Aβ-induced effects was evaluated using the JC-1 kit, terminal deoxynucleotidyl transferase dUPT nick end labeling, and western blotting. Gpc4 was stably expressed in NSCs, neurons, and astrocytes. Gpc4 was upregulated by Aβ in NSCs and regulated Aβ internalization. Gpc4 attenuation reduced Aβ uptake; Gpc4 overexpression increased Aβ uptake. Gpc4 regulated Aβ internalization through LRP1 and contributed to Aβ internalization and toxic/physical concentrations of Aβ-induced mitochondrial membrane potential and cell apoptosis, partly *via* LRP1. Therefore, Gpc4 is a key regulator of Aβ enrichment in NSCs. Inhibiting Gpc4 rescued the Aβ-induced toxic effect and attenuated the nontoxic Aβ enrichment into intracellular toxic concentrations. Gpc4 contributed to Aβ internalization and toxic/physical concentrations of Aβ-induced mitochondrial membrane potential damage and cell apoptosis, partly *via* LRP1. These findings suggest a potential role of Gpc4 in treating Alzheimer’s disease at an early stage, by targeting NSCs.

## Introduction

Neural stem cell (NSC) damage has been reported in the brains of Alzheimer’s disease (AD) patients (Wang et al., 2016; Huang et al., 2021). Cognitive decline in AD patients may rise because differentiation of NSCs in the adult brain cannot compensate for neuronal loss.

How does impairment of neurogenesis occur in patients with AD? Intracellular β-amyloid peptide (Aβ) aggregation occurs before the extracellular deposition and plaque formation (Bloom, 2014; Tiwari et al., 2019). Intracellular accumulation disrupts the cytoskeletal structure and induces cell apoptosis (Mohamed and Posse de Chaves, 2011). Aβ also induces neuronal toxicity in the early stages of AD (Oakley et al., 2006). Therefore, understanding the intracellular effects of Aβ will be beneficial for identifying potential targets for AD treatment. Manipulation of Aβ transport into NSCs is a promising line of research for the development of AD therapies.

Heparan sulfate proteoglycans accumulate abundantly in conjunction with Aβ (Timmer et al., 2010; Fu et al., 2016), and are particularly present in senile plaques (Ghazale et al., 2018). For two decades, the mRNA expression of glypican 4 (Gpc4) has been known to be upregulated in the brains of patients with AD, but its role in the disease remains to be elucidated.

Gpc4 is widely expressed in NSCs, astrocytes, and neurons. Its expression pattern suggests it has multiple roles during nervous system development. Interestingly, the heparan sulfate on the extracellular surface of Gpc4 has a high affinity for Aβ and its receptors, such as low-density lipoprotein receptor-related protein 1 (LRP1) and apolipoprotein E (ApoE; Liu et al., 2013, 2016). This suggests Gpc4 may play an important role in Aβ internalization in NSCs.

In this study, we hypothesized that Gpc4 may assist in Aβ internalization in NSCs, reducing their viability and facilitating the induction of apoptosis. Our results suggest this may be the case in the early stages of AD. Manipulating the transportation of Aβ into NSCs at an early stage by interfering with Gpc4 is a promising approach for AD therapeutic studies.

## Materials and Methods

All animal experimental protocols were devised in accordance with the National Institute of Health Guide for the Care and Use of Laboratory Animals and approved by the Institutional Animal Care and Use Committee at Xi’an Jiaotong University and. All efforts were made to minimize the number of animals used and their suffering.

### NSC Culture

NSCs were isolated from mouse embryos (E14.5) as previously described (Zhang et al., 2015; Jiao et al., 2016; Ghazale et al., 2018). Epidermal growth factor, fibroblast growth factor, B27, and N2 were added to Dulbecco’s Modified Eagle Medium/Nutrient Mixture F-12 as the NSC culture medium. NSCs from the P2 generation were plated at a density of 9 × 10^7^ cells/well. NSCs were transfected with Gpc4 siRNA adenovirus (AdV), according to the manufacturer’s instructions (GeneChem, Shanghai, China). NSCs were treated with AdV for 24 h and then the medium with AdV was changed to complete medium without AdV. After 72 h of infection, Aβ was added. Internalization was detected after 6 h, and toxic effects were detected after 12 h.

### Cell Viability Assay

Cell viability was evaluated using the Cell Counting Kit-8 (CCK-8, Sigma-Aldrich, St. Louis, MO, USA) assay. Cells were plated in 96-well plates at 20,000 cells/well density, 24 h before the experiments. The cells were then treated with different concentrations of Aβ (0, 200 nM, 1 μM, 2 μM, 5 μM, 10 μM, and 20 μM) for 12 h. Next, 20 μl of CCK-8 were added to each well and the cells were incubated for 2 h at 37°C. Absorbance was measured at 490 nm using a microplate spectrophotometer (BioTek, Winooski, VT, USA). Triplicate parallel wells were examined in each experiment, and data were collected from three independent experiments. The results are presented as a percentage of the absorbance compared to the control group.

### Terminal Deoxynucleotidyl Transferase dUPT Nick End Labeling (TUNEL) Staining

To investigate the toxic effect of Aβ on NSC apoptosis mechanism, a terminal deoxynucleotidyl transferase dUPT nick end labeling (TUNEL) assay was performed according to the manufacturer’s instructions (Roche Diagnostics, Basel, Switzerland). Cells were fixed on cover slides in 4% paraformaldehyde for 15 min. The cells were then permeabilized in 0.3% Triton X-100 in phosphate-buffered saline for 5 min on ice. Cells were incubated in the TUNEL reaction mixture for 1 h at 37°C. Subsequently, the cells were counterstained with 4′,6-diamidino-2-phenylindole (DAPI; 1:3,000, Sigma-Aldrich) before mounting. Images were taken within 2 h. Nine random fields were counted for each group with a 40× objective. The ratio was calculated as the percentage of TUNEL-positive cells compared to that of DAPI-positive cells.

### Flow Cytometry Analysis

Flow cytometry analysis was used for the apoptosis tests in this study. The propidium iodide (PI)/Annexin V apoptosis detection kit (BD Biosciences, Franklin Lakes, NJ, USA) was used according to the manufacturer’s instructions. Cells were dissociated, washed in cold phosphate-buffered saline, and resuspended in 1× binding buffer to obtain a concentration of 1 × 10^6^ cells/ml. The cells were incubated with PI/Annexin V for 15 min in the dark. Subsequently, the cell solution was analyzed using FACSCalibur (BD Biosciences) for PI/Annexin V. The percentage of apoptotic cells in this experiment was calculated as the ratio of Annexin V-positive and PI-negative cells from the lower right quarter.

### Immunohistochemistry

For immunohistochemistry performed in APP/PS1 mice, mouse brains were fixed with 4% paraformaldehyde and frozen coronal slices (30 μm) were obtained using a frozen section machine (Leica, Wetzlar, Germany). NSCs were fixed in 4% paraformaldehyde. The sections were treated with 0.3% Triton for 30 min. The cells were then blocked with 5% bovine serum albumin for 30 min. The sections were incubated with primary antibodies overnight at 4°C. The cells were then incubated with the secondary antibodies, goat-anti-rabbit-IgG-488 and goat-anti-mouse-IgG-594 (Invitrogen, Carlsbad, CA, USA), for 2 h at room temperature. Slides were cover-slipped using a mounting medium containing DAPI (blue). The primary antibodies used in this experiment were: mouse polyclonal anti-nestin antibody (1:200, Novus, St. Louis, MO, USA), rabbit polyclonal anti-Gpc4 antibody (1:100, Abcam, Cambridge, UK), rabbit polyclonal anti-sox2 antibody (1:200, Abcam), rabbit polyclonal anti-Ki67 antibody (1:200, Abcam, Cambridge, UK), mouse polyclonal anti-sox2 antibody (1:200, Abcam, Cambridge, UK), mouse polyclonal anti-LRP1 antibody (1:1,000, Abcam, Cambridge, UK), goat-anti-rabbit-IgG-488, goat-anti-mouse-IgG-594 (Invitrogen, Carlsbad, CA, USA), and DAPI (1:3,000, Sigma-Aldrich, USA). For 3,3’-diaminobenzidine staining, we followed the manufacturer’s instructions using a secondary antibody staining kit (ZSGB-BIO Inc., Beijing, China).

### Western Blotting

NSCs were homogenized in radioimmunoprecipitation assay buffer supplemented with a protease inhibitor and centrifuged (15,000× *g*) for 30 min at 4°C. The protein concentration was quantified using the BCA assay (Beyotime, Nanjing, China). Protein (10 μg) was added to each sample, electrophoresed on 10% sodium dodecyl sulfate polyacrylamide gels, and transferred to a polyvinylidene fluoride membrane (Millipore, Burlington, MA, USA). The membranes were then blocked in 10% skimmed milk for 2 h and incubated with the following primary antibodies: rabbit anti-Gpc4 (1:2,000), rabbit anti-Cyt c (1:500), rabbit anti-Bax (1:500), rabbit anti-Bcl 2 (1:500), rabbit anti-GAPDH (1:10,000), or β-actin (1:10,000) (Abcam, Cambridge, UK). The primary antibody was dissolved in 10% skimmed milk, and the membrane was incubated at 4°C overnight. The membrane was then washed and incubated with horseradish peroxidase-conjugated secondary antibody. Membranes were developed using enhanced chemiluminescence and exposed to photographic films. All results were obtained from three independent experiments. The results were analyzed using ImageJ software (Yang et al., 2015).

### Virus Infection

siGpc4, overexpressed Gpc4 (overGpc4), and siLRP1 were coated with AdV (4 × 10^12^ μg/ml) to infect NSC cell cultures. The overGpc4 sequence was obtained from Gene_ID:14735, GenBank: NM_008150. The forward siGpc4 sequence was 5-GGAUGGCAGU GGAUGACUUTT-3 and the reverse was 5-AAGU CAUCCACUGCCAUCCTT-3. The forward and reverse siLRP1 sequences were 5-GCCCAUUGGAUGAGUUUCATT-3 and 5-UGAAACUCAUCCAAUGGGCTT-3, respectively. The lentivirus vector for Gpc4 overexpression was GV367 Ubi-MCS-SV40-EGFP-IRES-pruromycin (GENE, Shanghai, China), and the lentivirus vector for siGpc4 was hU6-MCS-Ubiquitin-FGFP-IRES-puromycin (GENE, Shanghai, China). AdV was plated into 6-well plates at a density of 2 × 10^6^ cells/ml; 10 μl of 2 × 10^12^ μg/ml AdV was added to each well in NSC complete medium (GENE, Shanghai, China). Twenty-four hours after infection, the medium was replaced with a complete medium without AdV. The cells were used for the experiment 72 h after infection.

### Enzyme-Linked Immunosorbent Assay (ELISA)

10 μl of 2 × 10^12^ μg/ml AdV (overGpc4 and si Gpc4) was plated into 6-well plates at a density of 2 × 10^6^ cells/ml (GENE, Shanghai, China). Twenty-four hours after infection, the medium was replaced with a complete medium without AdV. Aβ was added into each 6-well plates cells at a working concentration of 200 nM or 2 μM, 72 h after Adv infection. NSC samples were collected 12 h after Aβ treatment. Mouse Aβ enzyme-linked immunosorbent assay (ELISA) Kit (R&D Systems, Minneapolis, MN, USA; Ma et al., 2016) and quantification experiments were conducted according to the manufacturer’s instructions (R&D Systems, Minneapolis, MN, USA). Protein samples in each group were detected three times in the 96T ELISA kit. Aβ concentrations were calculated by the standard curve. This experiment was repeated three times.

### APP/PS1 Mice

The APP/PS1 mice used in this study were double transgenic mice bred by crossing two lines of commercial simple transgenic mice: APPSWE (Tg2576 on a B6J background) and PS1dE9 on a C57BL6SJL background. Acute crossing of these two lines produces an accelerated mouse model of AD on a B6J/B6SJL background combining cognitive and amyloid pathologies starting at 4 months old. Genotypes were confirmed by a polymerase chain reaction from a tail biopsy. All wild type mice in this study are of B6J/B6SJL littermates. All APP/PS1 mice were APP-and PS1 positive. The animals were obtained by Yang Wei-Na from Xi’an Jiaotong University, housed in an animal facility with two mice per cage in temperature- (22 ± 1°C) and humidity-controlled (50 ± 10%) conditions under a 12 h light/dark cycle. The mice had access to food and water *ad libitum* for 4 months before being used in the experiment.

Gpc4 siRNA lentivirus was injected into each mouse’s brain *via* stereotaxic surgery under deep isoflurane anesthesia (*n* = 6). Gpc4 siRNA lentivirus was injected using stereotaxic coordinates and a microsyringe. Virus was injected into the lateral ventricles and the CA1 hippocampal subfield (rostrocaudal: 1.0 mm, mediolateral: 1.30 mm, dorsoventral: 1.8 mm vs. Bregma) of both sides (rostrocaudal: 1.80 mm, mediolateral: 1.00 mm, dorsoventral: 3.10 mm vs. Bregma). The injection speed was 0.2 μl/min, and the total volume/side was 1 μl, 2 × 10^8^/μl. The control virus was also injected into APP/PS1 mice (*n* = 5). After surgery, the animals were given 2 months for recovery and lentivirus expression.

### Thioflavin-S Staining

After the brain was perfusion-fixed and sectioned at 30 μm on a freezing microtome, the selected series were mounted onto gelatine-coated glass slides and air-dried. The tissue was hydrated by passing it through a series of ethyl alcohol solutions (100%, 95%, 80%, and 70%, 1–2 min in each) before staining with 1% Thioflavin-S for 30 min (Oakley et al., 2006). The slides were subsequently rinsed in distilled water and transferred through 80% and 95%, and two sets of 100%, ethanol for 30 s before cover-slipping with mounting media and storage under refrigeration. Thioflavin-S staining was viewed under a green fluorescence microscope (480/525 nm).

### Immunoprecipitation Experiment

Immunoprecipitation (IP; Proteintech, Chicago, IL, USA) was performed as previously described (Ma et al., 2018). Eluted proteins that bind to any protein complex containing Gpc4 were separated *via* sodium dodecyl-sulfate polyacrylamide gel electrophoresis. Gpc4, LRP1, and Aβ were detected using western blotting. Briefly, APP/PS1 mouse brain tissue supernatants containing 4 mg protein (in approximately 1 ml lysate) were pre-cleared for 1–4 h at 4°C with 100 μl of 80–100 protein G. Precleared supernatants were incubated overnight at 4°C with the desired IP antibody (MOAB2, 1:200; Gpc4, 1:100; goat anti-rabbit IgG, 1:100) and 200 μl of protein G beads. IP complexes were washed five times with wash buffer and eluted twice with 40 μl elution buffer. Protein complexes (80 μl) were obtained and used for western blotting.

### JC-1 for Mitochondrial Membrane Potential (MMP)

NSCs were stained with JC-1 to determine the mitochondrial membrane potential (MMP). JC-1 is a membrane-permeable lipophilic dye that exists as JC-1 aggregates in the mitochondrial matrix (red) or as monomers in the cytoplasm (green). During mitochondrial depolarization, the red JC-1 aggregates form green monomers due to a change in proton motive force (ΔΨ). Thus, depolarization can be measured as an increase in the green fluorescent/red fluorescent intensity ratio. The JC-1 assay was performed as follows. Solutions containing 5 μg/ml JC-1 were added for AdV and Aβ treated NSCs for 30 min. The stained cells were rinsed twice with the culture medium. After the fresh media was added, the cells were analyzed under a green fluorescence microscope (480/525 nm).

### Statistical Analyses

All data were analyzed with GraphPad Prism version 6.0 (GraphPad Software, San Diego, CA, USA). For normal distribution data, one-way analysis of variance (ANOVA) followed with Tukey’s multiple comparisons test were performed. *P*-value <0.05 was regarded as significant. *t-test* was used to compare between two groups. All *in vitro* experiments have been independently repeated at least three times. For *in vivo* experiments, in each group at least five mice were used for statistical analysis.

## Results

### Gpc4 Is Expressed in NSCs

**Figure 1:**
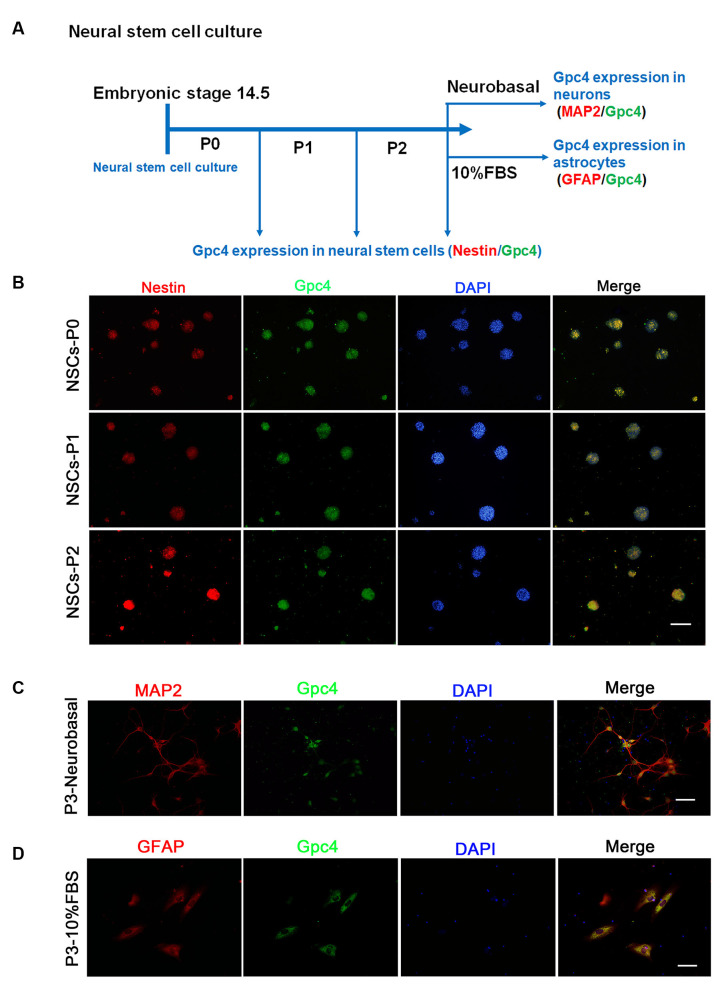
Expression of Gpc4 in neural stem cells (NSCs). **(A)** The schedule for detecting Gpc4 expression in NSCs and differentiated neurons and astrocytes. **(B)** At P0, P1, and P2, Gpc4 (green) colocalized with nestin (red) in the neural spheres. **(C,D)** NSCs were differentiated using neural basal medium containing B27 or DMEM/F12 containing 10% FBS. MAP2 (red) and GFAP (red) were used to illustrate Gpc4 (green) expression in neurons and astrocytes, respectively. Gpc4, glypican 4; DMEM/F12, Dulbecco’s Modified Eagle Medium/Nutrient Mixture F-12.

Neural stem cells derived from the cortex of embryonic 14.5 (E14.5) mice were observed. Approximately 5 days after primary culture (P0), the isolated NSCs were passed for the next generation (P1) and then for P2 (Figure 1A). We observed that neural spheres from P0-P2 stably expressed Gpc4 and nestin (Figure 1B). After NSC differentiation, we also detected high colocalization of Gpc4 and both neurons and astrocytes (Figures 1C,D).

### Different Concentrations of Aβ Affect NSC Viability and Apoptosis

**Figure 2:**
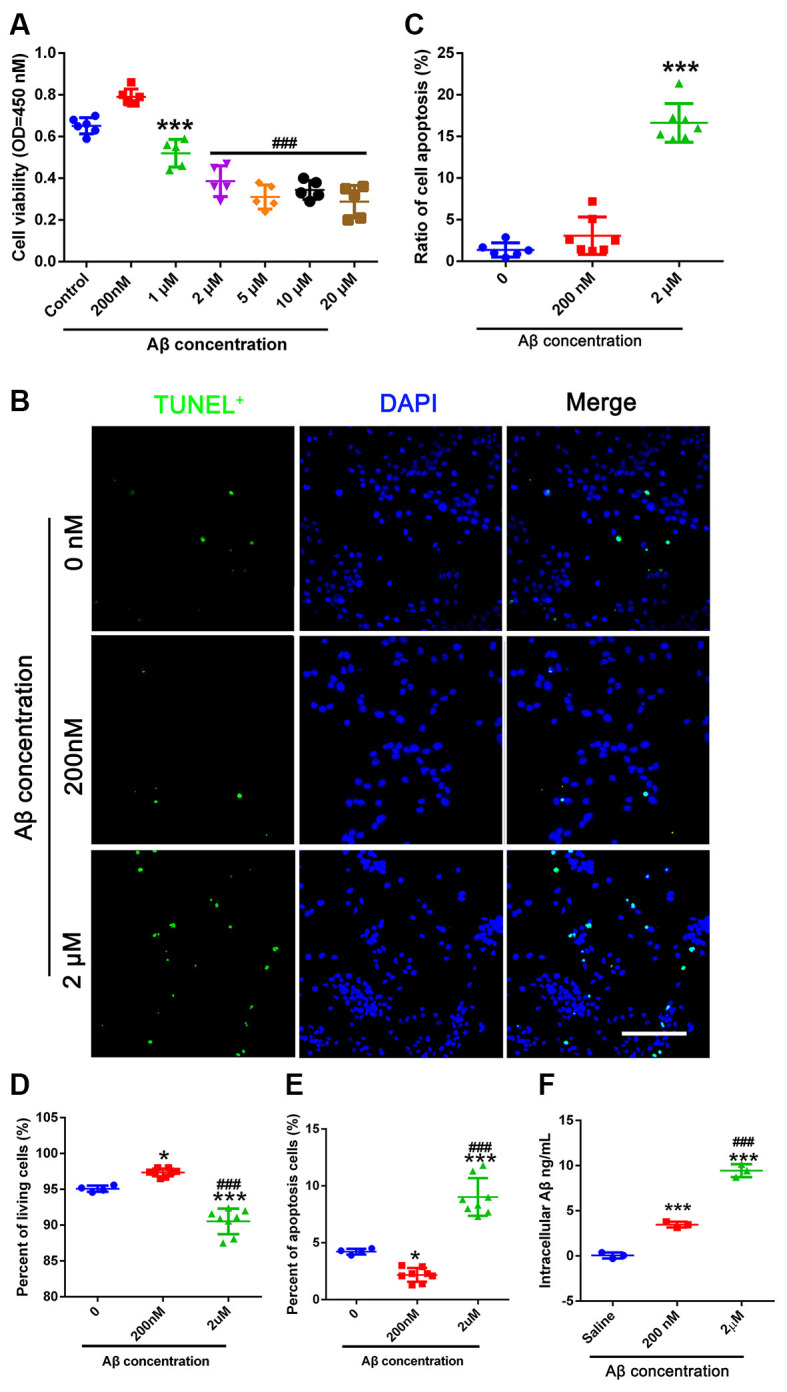
Different concentrations of Aβ affect NSC apoptosis. **(A)** CCK-8 was used to detect cell viability. NSCs were passed for 2–4 generations and plated into 96-well plates. Different concentrations of Aβ oligo were added for 24 h before the CCK-8 test. *** represents 1 μM vs. control group, *P* = 0.011. ^###^ represents 2–20 μM vs. control group, *P* < 0.0001. **(B)** TUNEL was used to obtain apoptotic cell numbers in 0 μM Aβ-, 200 nM Aβ-, and 2 μM Aβ-treated NSCs. **(C)** The ratio of TUNEL (green) positive cells compared to DAPI (blue) was calculated. *** represents 2 μM Aβ vs. 0 μM Aβ, *P* < 0.0001. Scale bar = 50 μm. **(D)** NSCs treated with 0 μM Aβ, 200 nM Aβ, and 2 μM Aβ were harvested 24 h after Aβ treatment and stained with PI/AnnexinV for flow cytometry analysis. The percentage of living cells was calculated. * represents 200 nM Aβ vs. 0 μM Aβ, *P* = 0.0181, *** represents 2 μM Aβ vs. 0 μM Aβ, *P* < 0.0001, ^###^ represents 2 μM Aβ vs. 200 nM Aβ, *P* < 0.0001. **(E)** Cells were treated as mentioned in **(D)**. The percentage of apoptotic cells was calculated. * represents 200 nM Aβ vs. 0 μM Aβ, *P* = 0.0236, *** represents 2 μM Aβ vs. 0 μM Aβ, *P* < 0.0001, ^###^ represents 2 μM Aβ vs. 200 nM Aβ, *P* < 0.0001. **(F)** 0 μM, 200 nM, and 2 μM Aβ were added to NSCs for 6 h. Intracellular Aβ was detected using the Aβ_1–42_-ELISA kit. *** represents 200 nM Aβ vs. 0 μM Aβ, *P* = 0.0003, *** represents 2 μM Aβ vs. 0 μM Aβ, *P* < 0.0001, ^###^ represents 2 μM Aβ vs. 200 nM Aβ. NSC, neural stem cells; CCK-8, cell counting kit 8; TUNEL, terminal deoxynucleotidyl transferase dUPT nick end labeling; DAPI, 4^′^,6-diamidino-2-phenylindole; PI, propidium iodide; ELISA, enzyme-linked immunosorbent assay.

This experiment aimed to determine the toxic dose of Aβ in NSCs. We found that 200 nM Aβ induced cell viability of NSCs, but that 1–20 μM Aβ was toxic to NSCs (Figure 2A). Therefore, 200 nM Aβ and 2 μM Aβ were used in this study. TUNEL staining showed that the number of apoptotic cells increased in the group treated with 2 μM Aβ compared to the 0 μM Aβ group (Figures 2B,C). We then used flow cytometry to detect PI/AnnexinV stained cells. The percentage of living cells in the group treated with 200 nM Aβ was significantly higher than that in the control group (0 μM Aβ). The percentage of living cells in the 2 μM Aβ group was significantly lower than that in the control group (0 μM Aβ). The apoptotic and living cell percentage in different concentrations of Aβ on NSCs is contrasting (Figures 2D,E). We used ELISA to detect the intracellular Aβ level. NSCs in the 200 nM group absorbed more Aβ than those in the control group. Cells treated with 2 μM Aβ showed a higher uptake of Aβ compared to the other groups (Figure 2F).

### Aβ Upregulates Gpc4 Expression in NSCs

**Figure 3:**
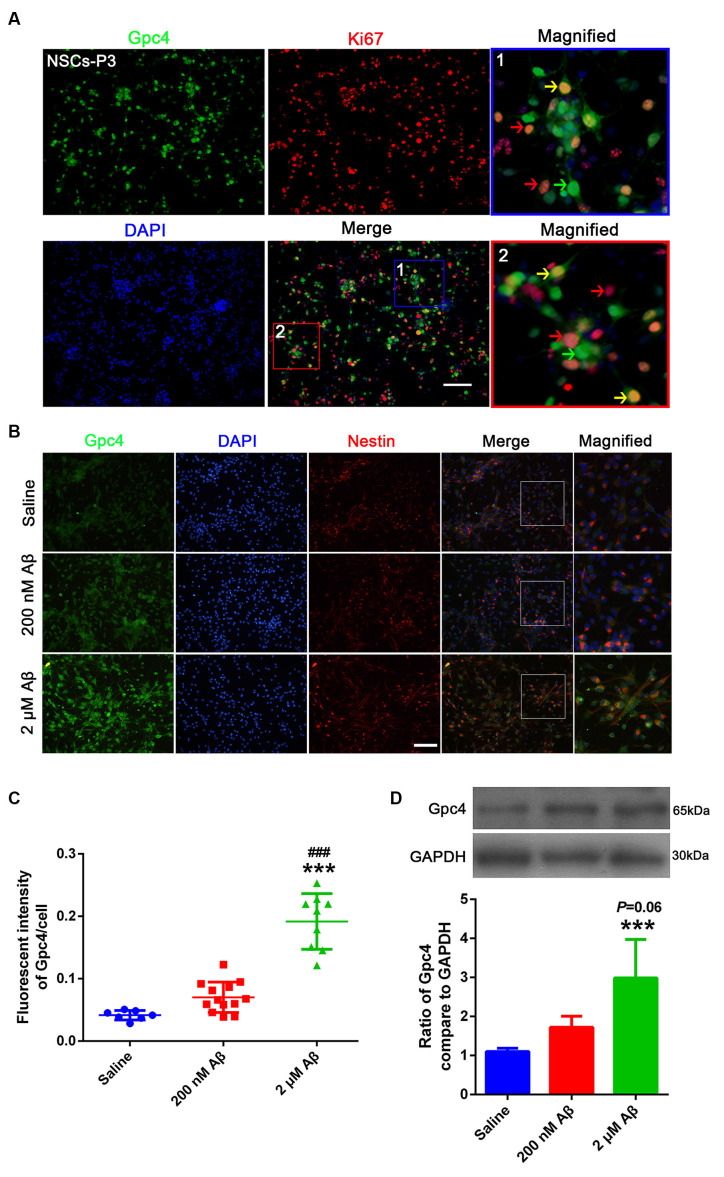
Aβ upregulated Gpc4 expression in NSCs. **(A)** Immunofluorescence analysis revealing the protein expression of Gpc4 (green) with Ki67 (red) in NSCs. Nuclei were counterstained with DAPI (blue). Gpc4 was widely expressed in NSCs (green). Double positive Gpc4/Ki67 NSCs (yellow arrow), Gpc4-negative Ki67-positive NSCs (red arrow), and Gpc4-positive Ki67-negative NSCs (green arrow) were also observed. Scale bar = 50 μm. **(B,C)** NSCs were treated with 0, 200 nM, and 2 μM Aβ for 24 h. Immunofluorescence analysis revealed the expression of Gpc4 (green), nestin (red), and DAPI (blue). Gpc4 colocalized with nestin-positive NSCs. *** represents 2 μM Aβ vs. saline, *P* < 0.0001, ^###^ represents 2 μM Aβ vs. 200 nM Aβ, *P* < 0.0001. Scale bar = 50 μm. **(D)** NSCs were treated with 0, 200 nM, and 2 μM Aβ for 24 h before western blotting. Western blotting was used to detect Gpc4 (56 kDa) and GAPDH (35 kDa) expression levels. *** represents 2 μM Aβ vs. saline, *P* = 0.007. Gpc4, glypican 4; NSCs, neural stem cells; DAPI, 4^′^,6-diamidino-2-phenylindole; GAPDH, glyceraldehyde 3-phosphate dehydrogenase.

To identify the role of Gpc4 in NSCs in AD, we examined Gpc4 expression patterns in NSCs. Gpc4 (green) colocalized with cells positive for Ki67 or nestin (red), which proves the expression of Gpc4 in NSCs. Gpc4 was widely expressed in NSCs. Double-positive Gpc4/Ki67 represents NSCs undergoing cell division (Figure 3A, yellow arrow). We also observed some Gpc4-negative Ki67-positive NSCs (red arrow) and Gpc4-positive Ki67-negative NSCs (green arrow). Furthermore, we assessed whether the expression of Gpc4 was regulated by Aβ treatment. NSCs were incubated with 200 nM and 2 μM Aβ for 12 h. Our results from immunofluorescence staining and western blotting illustrated that Gpc4 expression increased with Aβ concentration (Figures 3B–D).

### Gpc4 Regulates NSC Uptake of Aβ

**Figure 4:**
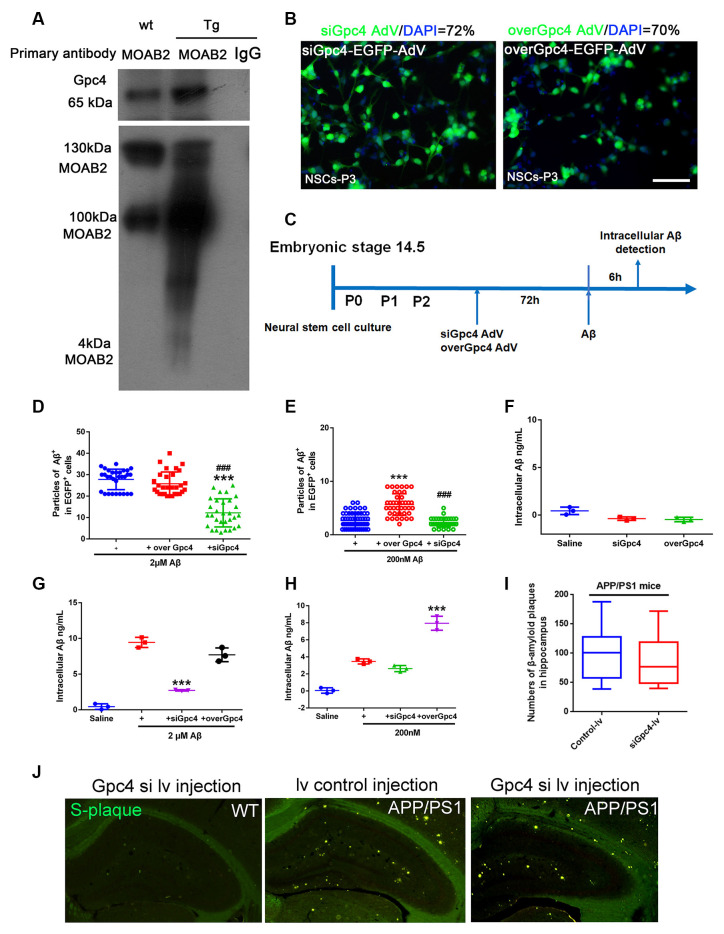
Gpc4 regulates Aβ internalization in NSCs. **(A)** Co-immunoprecipitation result from protein lysis extracted from 6-month-old APP/PS1 mice. Protein samples were incubated with the primary antibody MOAB2. Protein complexes able to bind to MOAB2 (Aβ42) were eluted. Western blotting was used to detect MOAB2 (Aβ42) and Gpc4 expression. **(B)** NSCs were plated in complete medium, and 2×10^12^ siGpc4 and overGpc4 AdV expression using enhanced green fluorescent protein (green) was added for 24 h. The medium was changed to complete medium, and cells were incubated for 48 h. Nuclei were marked with DAPI (blue). The ratio for AdV infection was calculated. **(C)** The schedule of virus infection and Aβ treatment on NSC cells. Seventy-two hours after virus infection, Aβ was added. Six hours after adding cy3-Aβ, all particles in AdV-positive cells were counted and compared among groups. **(D)** Six hours after adding 2 μM cy3-Aβ, all particles in AdV-positive cells were counted and compared among groups. *** represents siGpc4+ 2 μM Aβ vs. 2 μM Aβ, *P* < 0.0001, ^###^ represents siGpc4+ 2 μM Aβ vs. overGpc4+ 2 μM Aβ, *P* < 0.0001. **(E)** Six hours after adding 200 nM cy3-Aβ, all particles in AdV-positive cells were counted and compared among groups. *** represents overGpc4+ 200 nM Aβ vs. 200 nM Aβ, *P* < 0.0001, ^###^ represents siGpc4+ 200 nM Aβ vs. overGpc4+ 200 nM Aβ, *P* < 0.0001. **(F)** AdV was used to infect NSCs, and intracellular Aβ42 in NSCs was detected using ELISA. **(G)** NSCs were treated as previously described in **(C)**. Instead of cy3-Aβ, the Aβ42 were used in this experiment. Six hours after adding 2 μM Aβ42, Aβ42 levels were detected using ELISA. *** represents siGpc4+ 2 μM Aβ vs. 2 μM Aβ, *P* < 0.0001. **(H)** NSCs were treated as previously described in **(G)**. Six hours after adding 200 nM Aβ42, Aβ42 levels were detected using ELISA. *** represents overGpc4+ 200 nM Aβ vs. 200 nM Aβ, *P* < 0.0001. **(I,J)** siGpc4/overGpc4 lentivirus was injected into both lateral ventricles and the hippocampus of 4-month-old (APP/PS1) mice. Two months after lentivirus injection, mice were sacrificed for Thioflavin staining. β-amyloid plaques were stained in green. All particles were counted and compared against those in the hippocampus. NSCs, neural stem cells; Gpc4, glypican 4; AdV, adenovirus; DAPI, 4^′^,6-diamidino-2-phenylindole; ELISA, enzyme-linked immunosorbent assay.

Co-IP was performed to detect the relationship between Gpc4 and Aβ. We found that Gpc4 forms a protein complex with Aβ in the APP/PS1 mouse brain (Figure 4A). To further determine the role of Gpc4 in Aβ internalization, AdV and lentivirus were used to either overexpress or attenuate Gpc4 expression in NSCs and APP/PS1 mice, respectively. The AdV transfection ratio was 72% for siGpc4-AdV and 70% for overGpc4-AdV (Figure 4B). The timing for the experimental procedure of Gpc4 and LRP1 AdV and Aβ treatment is laid out in Figure 4C. Furthermore, cy3-Aβ (200 nM and 2 μM) was added to siGpc4-AdV-, Gpc4-AdV-, or control-AdV-infected NSCs for 6 h. Subsequently, the Aβ particles per cell were counted. Attenuating Gpc4 decreased the number of Aβ particles in the 200 nM and 2 μM groups. Overexpression of Gpc4 was able to upregulate the Aβ particle number in the 200 nM group but had no significant effect in the 2 μM group (Figures 4D,E).

Next, cells were monitored to detect whether Gpc4 induced Aβ production. No significant differences were observed between the siGpc4 and overGpc4 adenovirus groups (Figure 4F). overGpc4 had no significant effect on 2 μM Aβ internalization, but siGpc4 significantly attenuated the internalized Aβ level (Figure 4G). In the 200 nM Aβ-treated group, overGpc4-AdV increased NSC Aβ internalization, but siGpc4-AdV had no significant effect on blocking Aβ internalization (Figure 4H). siGpc4/overGpc4 lentivirus were injected into the hippocampus of 4-month old (APP/PS1) mice brains, and 2 months after injection, mice were sacrificed for S-plaque detection. Aβ plaques were stained using Thioflavin staining. Plaque numbers in the siGpc4 and overGpc4 groups did not change at this stage in APP/PS1 mice (Figures 4I,J). These results suggest an important dose-dependent effect of Gpc4 on Aβ internalization in NSCs at the early stage of AD.

### LRP1 Is Expressed in NSCs and Regulates the Aβ Internalization

**Figure 5:**
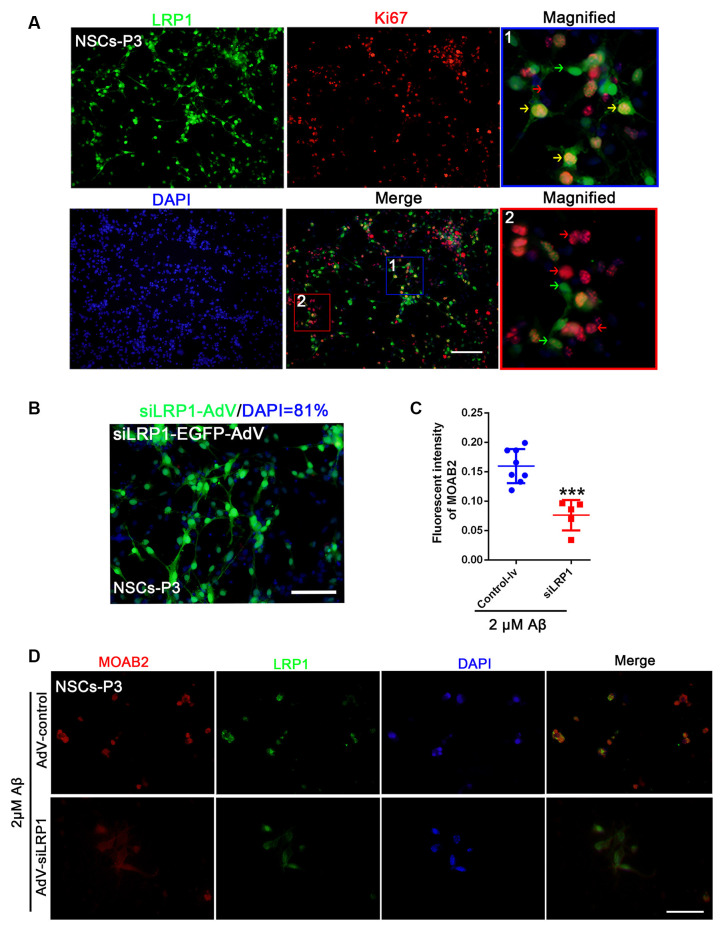
LRP1 is expressed in NSC and regulates the internalization of Aβ. **(A)** Immunofluorescence analysis revealing the expression of LRP1 (green) with Ki67 (red) in NSCs. Nuclei were counterstained with DAPI (blue). LRP1 was widely expressed in NSCs (green). Double positive LRP1/Ki67 (yellow arrow), LRP1-negative Ki67-positive NSCs (red arrow), and LRP1-positive Ki67-negative NSCs (green arrow) were also observed. Scale bar = 50 μm. **(B)** NSCs were plated in complete medium, and then siLRP1 adenovirus expression using enhanced green fluorescent protein (green) was added (2×10^12^) for 24 h for transfection. The medium was then changed for 48 h. Nuclei were marked with DAPI (blue). The ratio of adenovirus transfection was calculated. **(C,D)** NSCs were plated in complete medium, and then control-lentivirus and siLRP1 lentivirus were added 24 h for transfection. The medium was then changed for 48 h. Twelve hours after adding 2 μM Aβ, fluorescence intensities of MOAB2 and LRP1 were measured and compared. *** represents siLRP1+ 2 μM Aβ vs. 2 μM Aβ, *P* < 0.0001. LRP1, low-density lipoprotein receptor-related protein 1; NSCs, neural stem cells; DAPI, 4^′^,6-diamidino-2-phenylindole.

LRP1 is an Aβ receptor that plays a role in Gpc4-induced Aβ internalization. We first identified the expression of LRP1 in NSCs by double staining NSCs with Ki67 and LRP1. LRP1 colocalized with Ki67-positive cells (Figure 5A). Double-positive LRP1/Ki67 (yellow arrow), LRP1-negative Ki67-positive (red arrow), and LRP1-positive Ki67-negative NSCs (green arrow) were all observed (Figure 5A). Moreover, the transfection ratio of siLRP1 was 81% (Figure 5B. The effect of LRP1 on Aβ internalization was analyzed by double staining of LRP1 with the Aβ antibody MOAB2 in AdV control and AdV-siLRP1-treated groups. siLRP1 remarkably decreased Aβ levels in NSCs (Figures 5C,D), suggesting LRP1 is responsible for Aβ transportation in NSCs. However, the relationship between LRP1 and Gpc4 remains unclear.

### Gpc4 Regulates Aβ Internalization Partially *via* LRP1 in NSCs

**Figure 6:**
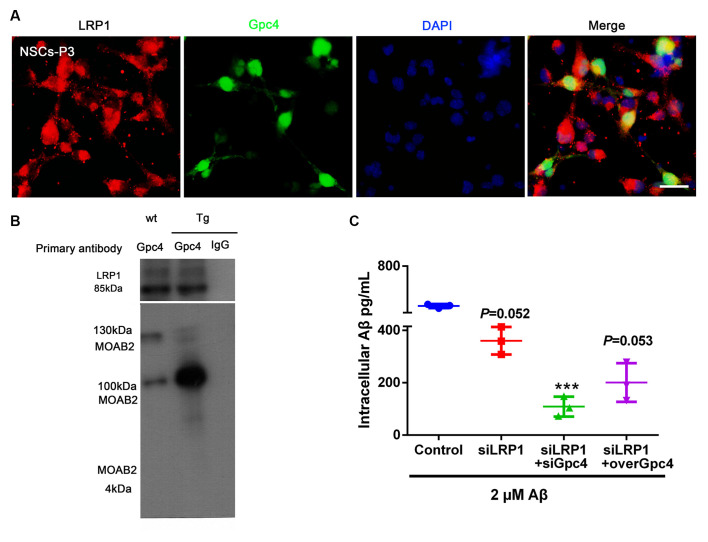
Gpc4 regulates Aβ internalization partially via LRP1 in NSCs. **(A)** Immunofluorescence revealed LRP1 (red) abundantly colocalized with Gpc4 (green) in NSCs. Nuclei were counterstained with DAPI (blue), scale bar = 20 μm. **(B)** Co-immunoprecipitation result from protein lysis extracted from 6-month-old APP/PS1 mice. Protein samples were incubated with primary antibody Gpc4. Protein complexes able to bind to Gpc4 were then eluted. Western blotting was used to detect the MOAB2- (Aβ42) and LRP1-positive binds. **(C)** Seventy-two hours after adenovirus transfection, 2 μM Aβ42 was added to the control, siLRP1, siLRP1+siGpc4, and siLRP1+overGpc4 groups. The levels of Aβ42 were detected using ELISA. *** represents siLRP1+ 2 μM Aβ vs. siGpc4+siLRP1+ 2 μM Aβ, *P* = 0.0011. GPc4, glypican 4; LRP1, low-density lipoprotein receptor related protein 1; NSCs, neural stem cells; DAPI, 4^′^,6-diamidino-2-phenylindole; ELISA, enzyme-linked immunosorbent assay.

To more precisely define the relationship between Gpc4 and LRP1, co-IP was performed on the cell lysis of APP/PS1 mice. Gpc4 was able to bind to LRP1, forming a protein complex in the brains of AD mice (Figures 6A,B). These observations suggest that LRP1 plays a role in Gpc4-induced Aβ internalization.

We next aimed to reveal the action of Gpc4 and LRP1 in Aβ internalization. NSCs were transfected with control, siLRP1, siLRP1+siGpc4, and siLRP1+overGpc4 AdV for 3 days, and then Aβ was added for 6 h before cells were harvested for ELISA. siLRP1 remarkably decreased Aβ uptake by NSCs. In the siLRP1 and siGpc4 groups, the amount of internalized Aβ was much less compared to the siLRP1-treated group. Furthermore, in the siLRP1+overGpc4-treated group, the internalized Aβ level was increased compared to the siLRP1+siGpc4 group (Figure 6C).

### Gpc4 Regulates Cell Apoptosis *via* LRP1 in NSCs

**Figure 7:**
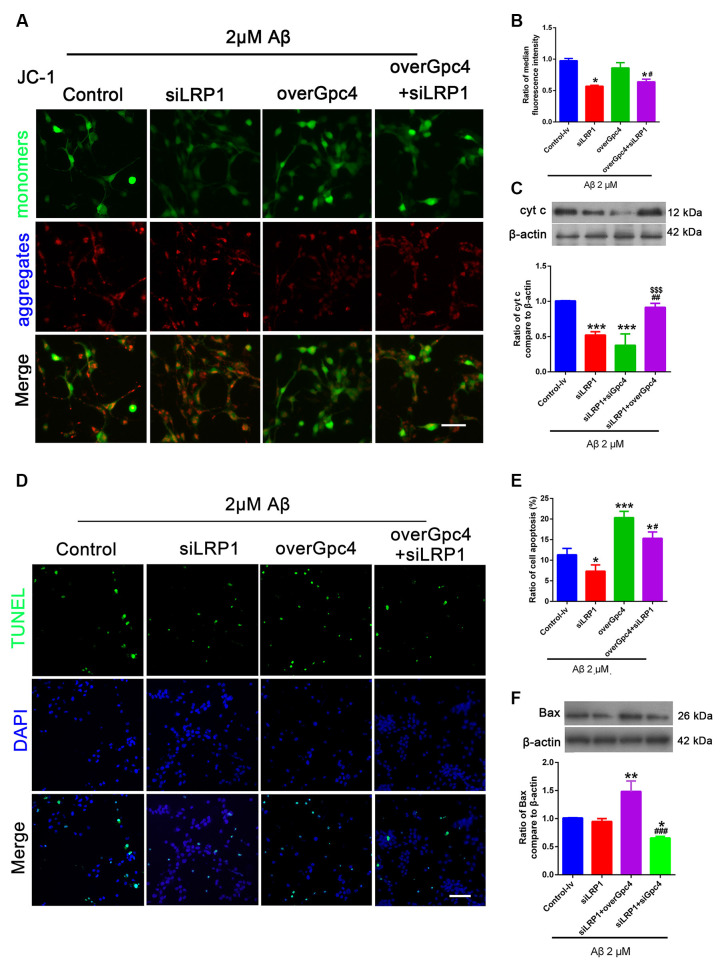
Gpc4 regulates cell apoptosis partly via LRP1 in NSCs. **(A,B)** NSCs were infected with control, siLRP1, overGpc4, and overGpc4+siLRP1 AdV for 3 days, and 2 μM Aβ was added for 12 h. The JC-1 kit was used to detect the mitochondrial membrane potential in NSCs. JC-1 monomers (green) and aggregates (red) were compared and normalized to the control group. The ratio of monomers to aggregates was calculated. **(C)** Seventy-two hours after AdV infection, 2 μM Aβ42 was added to the control, siLRP1, siLRP1+siGpc4, and siLRP1+overGpc4. The levels of Cytc were determined using western blotting. *** represents siLRP1+siGpc4 vs. control, *P* = 0.0001, siLRP1 vs. control, *P* = 0.0007; siLRP1+overGpc4 vs. siLRP1, *P* = 0.0028. ^$$$^siLRP1+siGpc4 vs. siLRP1+overGpc4, *P* = 0.0252, *siLRP1+siGpc4 vs. siLRP1+overGpc4, *P* = 0.0003. **(D,E)** NSCs were infected with control, siLRP1, overGpc4, and overGpc4+siLRP1 AdV for 3 days, and Aβ was added for 12 h. TUNEL staining was used to detect whether Gpc4 regulated Aβ-induced cell death via LRP1. The ratio of apoptotic cells was calculated based on TUNEL positive cells (green) and DAPI (blue). **(F)** The expression of Bax was also detected using western blotting, ** represents siLRP1+overGpc4 vs. siLRP1, *P* = 0.0007, *siLRP1+siGpc4 vs. siLRP1, *P* = 0.0252, *siLRP1+siGpc4 vs. siLRP1+ overGpc4, *P* < 0.0001. GPc4, glypican 4; LRP1, low-density lipoprotein receptor related protein 1; NSCs, neural stem cells; DAPI, 4^′^,6-diamidino-2-phenylindole; AdV, adenovirus; TUNEL, terminal deoxynucleotidyl transferase dUPT nick end labeling.

Control, siLRP1, overGpc4, and overGpc4+siLRP1 AdV were transfected into NSCs for 3 days, and then Aβ was added for 12 h. The JC-1 kit was used to detect the MMP in NSCs. JC-1 monomers (green) and aggregates (red) were compared and normalized to those of the control group. In 2 μM Aβ experiments, JC-1, TUNEL, and western blotting were used to determine whether Gpc4/LRP1 regulates the toxic effect of Aβ on NSCs. siLRP1 significantly decreased the ratio of JC-1 monomers/aggregates compared to the control group. Moreover, overGpc4 increased MMP damage compared to siLRP1. overGpc4+siLRP1 significantly increased the MMP compared to siLRP1; however, the MMP damage in the overGpc4+siLRP1 group was lower than that in the overGpc4 group (Figures 7A,B). Moreover, the level of Cytc was decreased in the siLRP1 and siLRP1+siGpc4 groups, and higher in the siLRP1+overGpc4 group (Figure 7C). Furthermore, TUNEL staining was used to detect whether Gpc4 and LRP1 regulate Aβ-induced cell death. Compared to the control group, siLRP1 attenuated NSC apoptosis while overGpc4 increased it. siLRP1 decreased the effect caused by overGpc4 (Figures 7D,E). The expression of Bax was also detected using western blotting, which is consistent with the results of TUNEL staining (Figures 7D–F).

**Figure 8:**
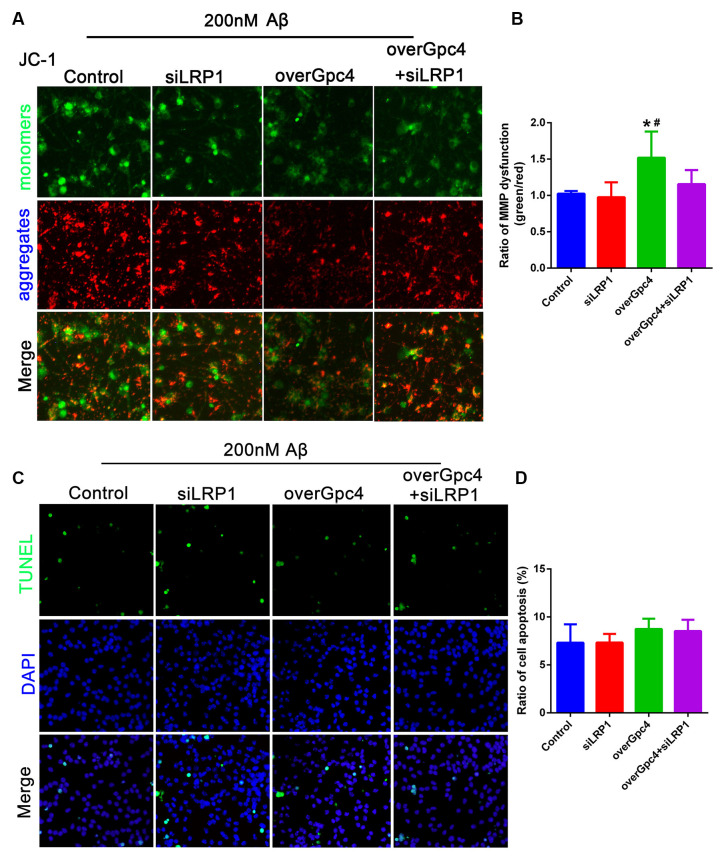
Role of Gpc4 and LRP1 on 200 nM Aβ on NSCs. **(A,B)** NSCs were infected with control, siLRP1, overGpc4, and overGpc4+siLRP1 AdV for 3 days, and then 200 nM Aβ was added for 12 h. The JC-1 kit was used to detect the mitochondrial membrane potential in NSCs. JC-1 monomers (green) and aggregates (red) were compared and normalized to those of the control group. The ratio of monomers/aggregates was calculated. * represents overGpc4 vs. control, *P* = 0.0036, ^#^ represents overGpc4 vs. siLRP1, *P* = 0.0080. **(C,D)** NSCs were infected with control, siLRP1, overGpc4, and overGpc4+siLRP1 AdV for 3 days, and 200 nM Aβ was added for 12 h. TUNEL staining was used to detect whether Gpc4 regulated the Aβ-induced cell death via LRP1. The ratio of apoptotic cells was calculated based on TUNEL positive cells (green) and DAPI (blue). NSCs, neural stem cells; GPc4, glypican 4; LRP1, low-density lipoprotein receptor related protein 1; AdV, adenovirus; TUNEL, terminal deoxynucleotidyl transferase dUPT nick end labeling; DAPI, 4^′^,6-diamidino-2-phenylindole.

In 200 nM Aβ experiments, JC-1 and TUNEL assays were used to determine whether Gpc4 induces toxic effects by enriching the nontoxic effect of Aβ on NSCs. Initially, 200 nM Aβ had no toxic effect on NSCs, but overGpc4 in 200 nM Aβ-treated NSCs increased MMP damage (Figures 8A,B). Furthermore, TUNEL staining was used to detect whether Gpc4/LRP1 regulated Aβ-induced cell death. No significant changes were observed among the four groups (Figures 8C,D).

## Discussion

The dysregulation of adult neurogenesis in AD occurs at an early stage of the disease and the mechanism has not been completely elucidated (Boese et al., 2020). Heparan sulfate proteoglycan, a highly conserved protein expressed in many species and tissues, is involved in basic cellular processes, such as cell proliferation, cell growth, axon guidance, and synapse formation (Rawson et al., 2005; Stewart and Sanderson, 2014; Jiao et al., 2016; Condomitti and de Wit, 2018), and is highly related to AD pathology. Twenty years ago, Gpc4, a heparan sulfate proteoglycan, was reported to be increased in the brains of AD patients and was initially found to be responsible for neurogenesis (Gysi et al., 2013; Blanchette et al., 2015; Zhang et al., 2015). This suggests that abnormal expression of Gpc4 in AD patients may contribute to NSC damage. We confirmed that Gpc4 is highly expressed in NSCs (Luxardi et al., 2007; Yu et al., 2017). Gpc4 expression was significantly higher in Aβ-treated NSCs than in control group NSCs. Gpc4 also regulated the internalization of Aβ by NSCs and enriched low concentrations of Aβ into NSCs. The overexpression of Gpc4 suggests that it may play a role in Aβ-induced toxicity in NSCs.

Heparan sulfate proteoglycans have been proposed to be responsible for Aβ enrichment. We found that Gpc4 expression was increased in Aβ-treated NSCs in a concentration-dependent manner. Nevertheless, considering neurogenesis deficiency is an important aspect of memory loss, Gpc4 may regulate Aβ enrichment in NSCs. The average concentration of Aβ42 in the brain white matter of patients with AD is 60 pM/L (Collins-Praino et al., 2014; Fu et al., 2016). While the concentration of Aβ in the brain extracellular fluid, such as the interstitial fluid and cerebrospinal fluid, is low (10^−10^–10^−9^ M; Ma and Qian, 2019), *in vitro* studies have suggested that the critical concentration for spontaneous aggregation of Aβ is in the μM range. Therefore, Aβ concentrations *in vivo* would have to be increased by at least three orders of magnitude for spontaneous aggregation. Several mechanisms have been proposed to explain this large concentration gap. The most likely hypotheses are that the concentration could be increased through membrane association (Gorbenko and Kinnunen, 2006; Aisenbrey et al., 2008) or macromolecular crowding (Munishkina et al., 2004; Hu et al., 2009; Fernandez-Perez et al., 2020; Wang et al., 2020; Wu et al., 2020). However, the mechanism underlying Aβ enrichment in NSCs remains unknown.

The role of Gpc4 in regulating different concentrations of Aβ internalization in NSCs is Aβ dose-dependent. siGpc4 decreased NSC uptake of 2 μM Aβ, and overGpc4 increased NSC uptake of 200 nM Aβ. This different role of Gpc4 on 2 μM Aβ and 200 nM Aβ transportation indicates that Gpc4 is functional at nanomolar Aβ concentrations; however, the transportation efficiency of Gpc4 reaches its saturation point at micromolar Aβ concentrations. Gpc4 is a key regulator of Aβ enrichment at nanomolar concentrations of Aβ. This illustrates why siGpc4 lentivirus had no effect on β-amyloid plaque numbers. Therefore, Gpc4 is a key regulator of Aβ enrichment during the early stages of AD, but not in the later stages.

Heparan sulfate, the extracellular portion of Gpc4, has a high affinity for Aβ and its receptors (Yamada and Hamaguchi, 2018; Du et al., 2020). Thus, heparan sulfate on Gpc4 may play a role in regulating Aβ internalization. Heparan sulfate also has a high affinity for LRP1 and the cellular prion protein (Taylor et al., 2009; Gao et al., 2019; Hu et al., 2019), which are also key receptors for Aβ transportation. This suggests that Gpc4 may gather the receptor and Aβ to the cell surface during the early stage of AD. However, the relationship between Gpc4 and the classic Aβ receptor LRP1 is yet to be determined.

LRP1 is an important receptor for the induction of Aβ internalization in astrocytes. Previous studies have shown that LRP1 clears 73.8% of Aβ injected into the brain towards blood circulation through the blood-brain barrier (Ma et al., 2018; Van Gool et al., 2019). Through the LRP1-related pathway, Aβ is internalized by neurons and localized in lysosomes, endosomes, and mitochondria, with little recycling (Lillis et al., 2005). ApoE lipid rafts (Gilat-Frenkel et al., 2014; Rushworth and Megson, 2014), the ERK1/2 pathway, Wnt3a, JNK, and other heparan sulfate proteoglycans may also play a role in Aβ internalization (Lai et al., 2010; Shi et al., 2011; Li et al., 2019; Mii and Takada, 2020). First, decreased LRP1 allowed more ApoE4-coated Aβ to pass through the NSC membrane *via* Gpc4 overexpression, leading to an upregulation of intracellular Aβ levels. Second, ERK1/2, as the downstream target of both LRP1 and Gpc4, may also regulate the expression of other Aβ receptors. siLRP1 decreased the expression of ERK1/2; however, Gpc4 overexpression activated ERK1/2, subsequently activating other receptors related to Aβ internalization. Lastly, Gpc4 is responsible for many cellular growth processes, such as cell division and cell metabolism (Farhy-Tselnicker et al., 2017; McGough and Vecchia, 2020). It is possible that overGpc4+siLRP1 may activate these processes and therefore upregulate Aβ transportation.

We have previously shown that internalized Aβ is transported into the mitochondria *in vitro* and *in vivo* (Yang et al., 2015; Ma et al., 2016, 2018). In this study, we found that Gpc4 regulates mitochondrial dysfunction and cell apoptosis in NSCs partly *via* LRP1. We used the JC-1 kit to detect the role of internalized Aβ on MMP, which is also called mitochondrial depolarization. We found that in toxic Aβ-treated groups, attenuating Gpc4/LRP1 expression decreased intracellular Aβ, MMP damage, and NSC apoptosis. In the physical Aβ-treated groups, Gpc4 overexpression caused MMP damage but had no effect on cell apoptosis. These results indicate that internalized Aβ is harmful to both mitochondrial function and cell viability.

## Conclusion

We demonstrated that Gpc4 regulates Aβ internalization at an early stage of AD. This process is dose-dependent, and occurs partly *via* the well-known Aβ receptor, LRP1. However, many questions remain unanswered. Heparan sulfate on the extracellular surface of Gpc4 is important for receiving and binding to Aβ and its receptors. Gpc4 was initially reported to have a major effect on synapse formation and neurogenesis; therefore, it is also important to determine whether Gpc4 mediates NSC-derived neurogenesis by regulating Aβ internalization in NSCs.

## Data Availability Statement

The original contributions presented in the study are included in the article, further inquiries can be directed to the corresponding author.

## Ethics Statement

The animal study was reviewed and approved by Institutional Animal Care and Use Committee at Xi’an Jiaotong University.

## Author Contributions

KM performed most of the experiments, analyzed data, and wrote the rough draft. SX performed experiments, data analysis, and redrafting. YLu and YiLi performed NSCs cell culture. CZ performed the animal housing. ZZ and YF took part in the virus infection experiment. ZZ and YoLi participated in the experiment design. XC designed the research and finalized the manuscript. All authors contributed to the article and approved the submitted version.

## Conflict of Interest

The authors declare that the research was conducted in the absence of any commercial or financial relationships that could be construed as a potential conflict of interest.

## Publisher’s Note

All claims expressed in this article are solely those of the authors and do not necessarily represent those of their affiliated organizations, or those of the publisher, the editors and the reviewers. Any product that may be evaluated in this article, or claim that may be made by its manufacturer, is not guaranteed or endorsed by the publisher.

## Abbreviations

AD: Alzheimer’s disease
AdV: adenovirus
ApoE: apolipoprotein E
CCK-8: Cell Counting Kit-8
DAPI: 4′,6-diamidino-2-phenylindole
ELISA: enzyme-linked immunosorbent assay
Gpc4: glypican 4
IP: immunoprecipitation
LRP1: low-density lipoprotein receptor-related protein 1
MMP: mitochondrial membrane potential
NSC: neural stem cells
PI: propidium iodide
TUNEL: terminal deoxynucleotidyl transferase dUPT nick end labeling.
